# Validation of the French Smoking Cessation Motivation Scale with French Smokers Using a Mobile App for Smoking Cessation

**DOI:** 10.3390/ejihpe12080082

**Published:** 2022-08-19

**Authors:** Luz Adriana Bustamante, Lucia Romo

**Affiliations:** 1 Laboratoire EA 4430-CliPsyd, Department of Psychology, University of Paris Nanterre, 92000 Nanterre, France; 2 Research and Development Department, Kwit SAS, 67000 Strasbourg, France; 3 Hôpital Raymond-Poincaré, Assistance Publique Hôpitaux de Paris, 92380 Garches, France; 4 Inserm U1018 CESP, University of Paris Sanclay, 91190 Gif-sur-Yvette, France

**Keywords:** self-determination theory, motivation, smoking cessation, scale validation, e-health, mobile app treatment

## Abstract

To tailor and predict the outcomes of smoking cessation treatment, it is essential to identify the nature of motivation, as it is the basis for long-term change in healthy behaviors according to self-determination theory (SDT). The purpose of this study is to examine the psychometric properties of the French Smoking Cessation Motivation Scale (F-SCMS). The factorial structure and the psychometric properties were assessed with French-speaking users who had started a 9-step preparation program through a mobile app for smoking cessation (*n* = 13,044). The results of the present study confirmed content validity (CFI = 0.905, SRMR = 0.045, RMSEA = 0.087) and good internal consistency (α = 0.86, ωh = 0.7, ωt = 0.89) with CFA. The convergent validity was very small, but there were highly significant positive correlations between the willingness and readiness to quit with integrated and intrinsic subscales (rs = 0.25–0.37, *p* < 0.001). The amotivation subscale significantly had no correlation with any degree of willingness (r = 0.01, *p* < 0.001), ability (r = 0.01, *p* < 0.001), and readiness to quit (r = 0.02, *p* < 0.001). This scale facilitates future research regarding the nature of motivation to quit smoking in the French-speaking population.

## 1. Introduction

Smoking cessation has long been a global public health priority, as tobacco use is attributed with several negative consequences for health and life quality [1]. Addressing smoking cessation through tobacco use surveillance and prevention policies (i.e., increase in bans and restrictions on public places, cigarettes pack prices, and proper therapeutical interventions) has contributed to a drastic reduction in tobacco consumption [2]. In France, tobacco consumption has significantly decreased in the general population between 2016, when 29.4% of citizens smoked, and 2020, when 25.5% of citizens smoked. The challenge ahead is to help those who are marginalized due to social inequalities [2]. Engagement with mobile applications would allow for them to have accessibility to care and information, and the possibility of transposing several proven effective therapeutic principles, thus increasing long-term abstinence [3,4].

Motivation is a concept that has been widely studied by different approaches as a key determinant of the engagement in a change process, and long-term behavioral changes [5]. In the context of smoking cessation in France, motivation was assessed by identifying: the stage of change according to Prochaska and DiClemente’s theory of change [6], the intensity of motivation (insufficient or high) with the Q-MAT scale [7], and with commonly used and comparable measures such as the identification of the reasons to change (health, family) [8], and the assessment of the willingness, ability, and readiness to change—commonly known as the WAR measures [9]. The 1990 Reasons for Quitting Questionnaire (RFQ) no longer has construct validity and was not available in French [10]. To date, there is no valid French measure based on self-determination theory (SDT) that determines the nature of motivation that drives smoking cessation.

The SDT framework bridges two other theories that seek to explain the direction that drives an action: humanistic and behavioral theory [11]. Vallerand explains that “all individuals have an innate or natural tendency to develop an increasingly elaborate and unified self, but the context in which the individual evolves may support or restrict the attempt to master and integrate experiences into a coherent self. Therefore, the degree of self-determination that initiates change and sustains it over time depends on the nature of the motivation, whether it is internal or contextual”. In other words, behavioral changes such as quitting smoking are not simply a dichotomous state of going from smoker to nonsmoker. This is, above all, an appropriation process of the new behavior and the adaptation of an appropriate environment that leads to the development of a unified version of the self. This process is defined as *internalization:* the new behavior is no longer driven by controlled motivation, but into an autonomous motivation [12].

The commitment, persistence, or performance of behavioral change can be explained in terms of the degree of *internalization*, which can range from none (amotivation) to completely internalized (intrinsic motivation) [5]. According to the literature, the more internalized the motivation is, the better the commitment to the change process, its long-term outcome, and general well-being [12]. SDT-based interventions seek to facilitate internalization through autonomous motivation and a sense of competence [13].

To identify the factors that favor behavioral change through new digital interventions, it is important to assess each user’s baseline level of internalization. SDT proposes six motivation constructs: amotivation, external, introjected, identified, integrated, and intrinsic motivation [5]. Amotivation (Amot) consists of the lack of intention to act or act passively. External (Exter) regulation is based on operant behaviorism in which a behavior is maintained only under an external contingency (reward or punishment), whereas introjected (Introj) regulation is under an internal contingency (boosting self-worth, and avoiding guilt and shame). When the individual is personally invested by accepting the value and importance of the behavior, it is regulated via identification (Inden). Engagement with the behavior lies with achieving a goal because it is perceived to be worthwhile. Integrated (Inter) regulation is the more autonomous type of extrinsic motivation that implies that the new behavior is no longer perceived as a goal to be achieved, but is a result of the harmony of deeply held values and beliefs. Lastly, intrinsic (Intris) fulfillment regulation is the most autonomous motivation, which means that the individual has internalized the value of their actions and drives satisfaction in the new behavior itself. Intrinsic motivation has three subcategories: motivation to know, to experience stimulation, and to accomplish [14].

As mentioned above, no French-language questionnaire exists that assesses each type of motivation proposed by SDT in the context of smoking cessation. Existing scales measuring the substance withdrawal treatment only include four of the six factors of the SDT framework, leaving behind the former intrinsic motivation construct. The Reasons for Quitting Smoking (RFQ) from 1990 leads to a four-factor model with two intrinsic dimensions (concerns about health and desire for self-control) and two extrinsic dimensions (immediate reinforcement and social influence) [15]. The Treatment Motivation Questionnaire (TMQ) scale that assesses engagement in alcohol withdrawal treatment resulted in a four-factor model: internal motivation (11 items), interpersonal help seeking (6 items), confidence in treatment (3 items), and external motivation (4 items) [16]. The most recent study, in 2007, of the treatment self-regulation questionnaire (TSRQ) also resulted in a four-factor model: autonomous (6 items), introjection (2 items), external (4 items), and amotivation (3 items) [13].

The aim of this study is to develop a French scale that assesses the nature of motivation according to the SDT framework for smoking cessation.

## 2. Materials and Methods

### 2.1. Scale Development

The F-SCMS builds on previous work on other intrinsic and extrinsic motivation scales for engagement in general behaviors (Guay, Blanchard, Vallerand, 1997–2003), various health behaviors [13], work, sports [14,17], therapy [9,18], and substance abstinence [13,16].

#### 2.1.1. Phase 1. Defining the Items

During the first stage of development of the F-SCMS scale, all items of each of the questionnaires mentioned above were listed with their respective validation coefficient. To be consistent with the SDT framework and the psychometric proprieties of other SDT scales, we assessed each motivation construct with three items and a 5-point scale ranging from “strongly agree” to “strongly disagree”.

An initial filtering was carried out based on the wording of each item (initiation vs. abandonment of a behavior). Indeed, most of the scales assess the motivation to perform a proactive behavior, whereas quitting smoking is a restrictive act. The second filter was performed based on the respective validation coefficients of each item of the scales presented above.

#### 2.1.2. Phase 2. Pilot Testing

A first pretest was conducted with the Typeform platform, and 60 French-speaking adults recruited via social networks. It included the F-SCMS scale and five other questions presented as follows: Are you currently in the process of changing your smoking behavior? How old were you when you started smoking? How old are you today? What gender do you identify with? How many cigarettes do you smoke per day?

According to Cronbach’s alpha, our first version did not have good internal consistency (0.57). The correlation between items was modest within the types of motivation except for intrinsic motivation.

#### 2.1.3. Phase 3. Item Modification

The 6 items that were poorly correlated with their assigned factor in the confirmatory factor analysis were reformulated. To reformulate the three amotivation items, interviews were conducted with two individuals who were currently not interested in quitting smoking. For the external item, the focus was instead on gaining time to perform another activity. For the item of introjected motivation, instead of saying only “because I feel guilty”, the item was rephrased into “I feel guilty when I do nothing to resolve my problems”. For the item of integrated motivation, instead of saying only “I want to improve my lifestyle”, the item was rephrased into “I choose to take better care of myself and my health”.

The Levesque and Boisvert scales were principally used as inspiration for the construction of this scale [13,18].

#### 2.1.4. Phase 4. Administration of the Final Version of the F-SCMS Scale

The questionnaire was administered to 15,210 French-speaking adults initiating a 9-step smoking cessation preparation program using a mobile app. Each step of the preparation program was composed of different activities on the basis of effective CBT techniques [19]. Once each activity is completed, the user has instant access to the next one. In Step 0, the objective is to increase awareness of one’s own values and motivations when committing to a change process (smoking cessation); this step is called “Defining what is important”. To this end, the F-SCMS was administered along with two other motivation measures: motives to quit and motivation level.

Users agreed to the terms and conditions regarding the use of their data for research purposes after downloading the mobile app, creating their account, and initiating a 9-step smoking cessation preparation program. The users could sign out of this agreement at any time with a simple email. The application does not require the gathering of demographic data for its proper function; therefore, in compliance with the current regulations for the user’s privacy protection, these data were not collected. The validation of the F-SCMS scale is part of a prospective study that aims to identify the determinants of mobile app use for smoking cessation in French users. The study protocol was validated by the local French ethics committee on 25 March 2021. This study is also in accordance with the ethical principles of the Declaration of Helsinki.

### 2.2. Measures

#### 2.2.1. Motivation

Three self-reported metrics of motivation were measured: motives to quit, motivational nature, and motivational level.

##### Motives to Quit

Motives to quit is the first measure, assessed by the following statement: My main raison to quit smoking is.... After this statement, users were asked to choose one of the five reason to quit: health, wellbeing, economy, family, and baby project. These options were in line with two reviews on the reasons to quit smoking [8,19,20].

#### French Smoking Cessation Motivation Scale (F-SCMS)

The scale was presented as part of a program for smoking cessation via a mobile app (Appendix A). After completing the scale, participants received personalized feedback based on the subcategory’s motivation to which they scored the highest. There were three steps to determining what should be displayed as feedback. First, we computed the average of each motivation (Amot, Ext, etc.). Second, we grouped each motivation into two categories. The first category (Cat1) was composed of the three most extrinsic forms of regulation: amotivation, external motivation, and introjected motivation. The second category (Cat2) was composed of the most internal motivations: identified, integrated, and intrinsic. The third step was the comparison of the average between the two categories (Cat1 and Cat2). On the basis of this result, the most valued category (Cat1 or Cat2) or the category closest to the internalized regulation (if there were equal scores within each category) was chosen. If the two categories (Cat1 and Cat2) had equal scores, so-called “mixed” feedback was proposed.

##### Motivational Level

The motivational level was assessed through a visual analog scale of three questions measuring the willingness, ability, and readiness to quit (WAR Scale). Users could move the cue from 1 (the minimal score) to 10 (the highest) for each of the following questions: “To what extent this change is a priority for you right now?”, “To what extent are you confident in your ability to change right now?”, and “To what extent do you feel ready to take action”?

#### 2.2.2. Engagement

The drop-out measure allows for a more objective trend to engage in the smoking cessation preparation program. User engagement was assessed through an event assessment of the program completion rate and the interstep completion rate. The interstep completion rate is the proportion of users who moved from the last activity of the initial step (S0) to the next one (S1). The program completion rate was defined by the proportion of users who completed the program from first use (S0) to the final inapp activity (S8) that went from the preparation phase to the action phase by defining a quit date.

### 2.3. Statistical Analysis

After performing routine descriptive statistics, we ran confirmatory factor analysis with the required sample size [20]. The six natures of motivation were considered to be latent factors, and the items related to each nature were their respective observed variables. We investigated common-fit indices (CFI, SRMR, RMSEA) to confirm the adequacy of the proposed factorial structure to the collected data [21].

The internal consistency of the scale was analyzed using Cronbach’s alpha as well as total, hierarchical and asymptotic Omega (a coefficient higher than 0.7 was considered to be acceptable) [22] Lastly, concurrent validity with the WAR scale was checked using Bravais–Pearson’s correlation. The analyses were performed with RStudio 1.4.1106 [23], and the Lavaan package [24].

## 3. Results

Between 16 August and 31 December 2021, 15,210 users started the program, of which 85% (*n* = 13,044) completed the motivational scale, the assessment of the main reasons to quit, and the motivational level to quit smoking. Table 1 shows the main reasons to quit by participants’ motivation towards smoking cessation. Overall, the main reasons to quit smoking were chosen in the following order: health (45%), money (23%), wellbeing (20%), family (7%) and child project (5%). Participants’ main motivation towards smoking cessation was intrinsic (58%), the most internalized. Each reason to quit had a prominent type of motivation. For health: introjected (51%) and mixed (50%); for money: amotivation (42%); for wellbeing: identified and intrinsic (22% each); for family: external (18%); and for child project (amotivation—10%).

### 3.1. Psychometric Proprieties of the Final Version of the F-SCMS Scale

#### 3.1.1. Factorial Validity

**Figure 1:**
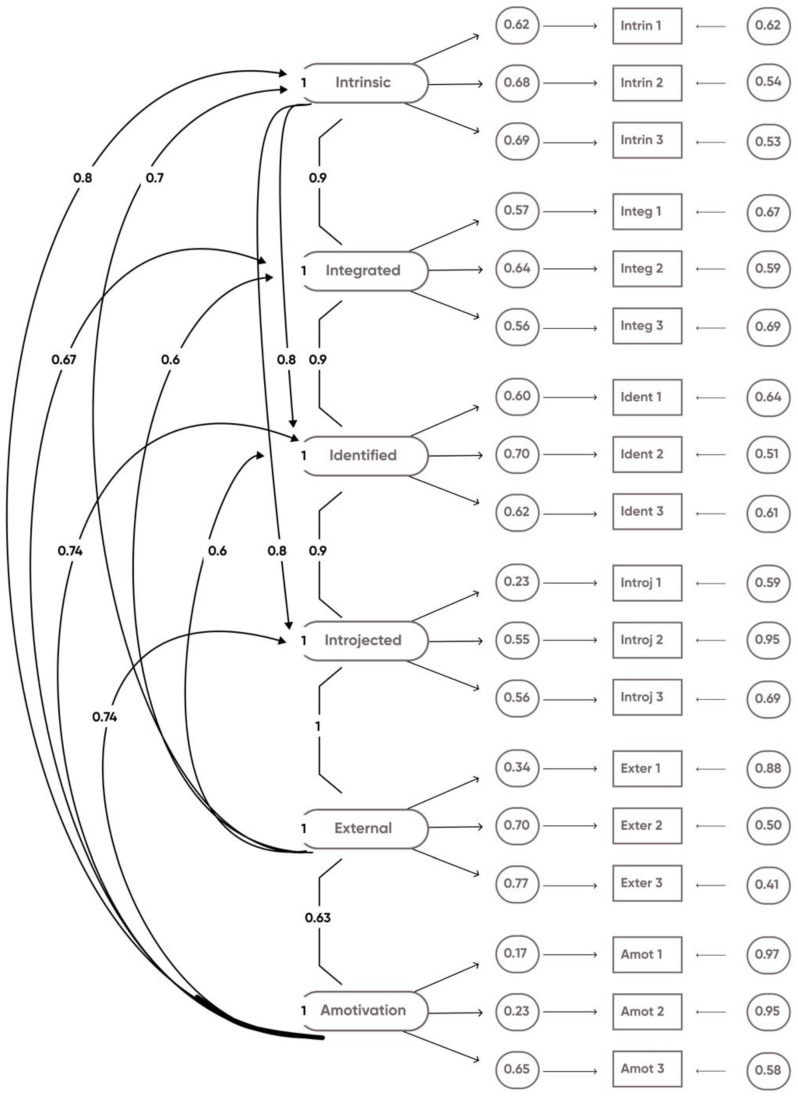
Confirmatory factor analysis of the six-factor structure of the French Smoking Cessation Motivation Scale (F-SCMS). Standarized solution; *n* = 13,044). All items were significantly regressed on their respective latent factors; *p* < 0.0000. Amot = amotivation; Exter = external; Introj = introjected; Ident = identified; Integ = integrated; Intrin = intrinsic.

We tested a model with three factors: amotivation (manifested by the three amotivation items), extrinsic motivation (manifested by the external, introjected, identified, and integrated motivation items), and intrinsic motivation (manifested by the three intrinsic motivation items). The CFA of this alternative model was CFI = 0.801, SRMR = 0.06, RMSEA = 0.087. The 6-factor model was more adapted with good-fit indices (CFI = 0.905, SRMR = 0.045, RMSEA = 0.064 CI 0.063–0.065). Figure 1 demonstrates that each of the 18 items significantly regressed on their respective latent factors with estimated ranges from 0.17 to 0.77 (all, *p* < 0.0000).

#### 3.1.2. Internal Consistency

The internal consistency of the scale was good according to Cronbach’s alpha (α = 0.86) and according to total (ωt = 0.89), hierarchical (ωh = 0.7) and h asymptotic (ω = 0.79) Omega. As shown in Table 2, these coefficients demonstrate acceptable internal consistency and support the reliability of the six motivation subscales (Amot = 0.80; Exter = 0.74; Introj = 0.74; Ident = 0.74; Iteng = 0.74; Integrated 0.78; Intrins = 0.73).

#### 3.1.3. Convergent Validity

Convergent validity was assessed with Pearson correlation analyses performed between the six subscales of the F-SCMS and the three items of the WAR scale. Table 3 shows generally very small but highly significant positive correlations among the willingness, ability, and readiness to quit with each subscale. Willingness to quit had small but significant positive correlation with identified (r = 0.39, *p* < 0.001), integrated (r = 0.37, *p* < 0.001), and intrinsic (r = 0.32, *p* < 0.001) regulation. Readiness to quit had small but significant positive correlation with integrated (r = 0.27, *p* < 0.001), and intrinsic (r = 0.25, *p* < 0.001) regulation. The amotivation subscale significantly had no correlation with any degree of willingness (r = 0.01, *p* < 0.001), ability (r = 0.01, *p* < 0.001) and readiness to quit (r = 0.02, *p* < 0.001).

#### 3.1.4. Concurrent Validity

Among the 15,211 users who filled in the motivation questionnaire, 5134 (34%) did not start the next step (S1) of the program. There was no clear trend as per dropout after the first step (S1) depending on the motivation’s nature. The interstep dropout rates per motivation (S1) were as follows: amotivation (*n* = 92, 35%), external (*n* = 371, 35%), introjected (*n*= 100, 34%), identified (*n* = 1218, 32%), integrated (*n* = 204, 36%), intrinsic (*n* = 2957, 34%), and mixed (*n* = 192, 41%). The program drop-out rate was extremely high ( *n* = 13,910, 91.5%) with no significant variations by internalized degree: amotivation (*n* = 242, 91.7%), external (*n* = 969 = 91, 7%), introjected (*n* = 274, 92.9%), identified (*n*= 3459, 90.7%), integrated (*n* = 516, 92.1%), intrinsic (*n* = 8015, 91.5%), and mixed (*n* = 435, 93.8%).

## 4. Discussion

Understanding motivational dynamics in smoking cessation is an important step in designing interventions to improve the engagement and effectiveness of the change process.

The aim of this paper was to develop a conceptually grounded measure based on self-determination theory (SDT) and examine the psychometric properties of the French Smoking Cessation Motivation Scale (F-SCMS). This scale was developed on the basis of other scales that measure the SDT framework in different domains, such as general substance use [13,16], treatment acceptance [9,18], and even scales that were not directly linked with substance consumption but have already considered the construct validity of internal motivation [14,17].

Consistent with previous work [9] and the hypothesis derived from the SDT motivation framework [5], internalized oriented participants were associated with greater readiness and willingness to quit smoking. Negative correlation would be expected between amotivation regulation and the WAR measure under the hypothesis that those with higher motivational regulation would be less ready, confident, and willing to quit smoking. On the contrary, the analysis showed that there was no correlation between these variables. These results can be explained by the presentation of each measure. The WAR scale is a visual analogical scale (VAS) composed by three direct questions of which the responses are likely to suffer from social desirability bias, whereas the result of the F-SCMS has a more complex scoring system.

The results of the present study confirm, through CFA, the convergent and internal validity of the F-SCMS with a six-factor structure representing the continuum of different motivations underlying the subscales of self-determination theory.

McCaul’s review of the main reasons to quit smoking highlighted the most important reasons: health (44–57%), social concerns (15–17%), and costs (12.5–14%). Each reason could be defined by other concepts. Health was related with health issues, whether they were actual or future concerns, illness in the social environment, or the willingness to feel better physically. Social concern was linked with social or familial pressure, or pregnancy [8]. The main reasons to quit in our sample were distributed as follows: health or wellbeing (65%), cost (23%), and family or child project (12%). In any case, according to self-determination theory, each reason for quitting smoking can be motivated by different types of regulation. This is illustrated by our results: those who chose wellbeing were mainly motivated by identified and intrinsic regulation, which means quitting smoking is perceived to be worthwhile and aligned with the deepest values of the person. Conversely, participants who chose health most often were those who were motivated by introjected regulation (avoiding guilt and shame). Family was the reason chosen by most of the participants with external regulation. They would like to earn time and money, and to be a source of pride within their families. When it comes to users who chose money and a child project as their primary reason, the main motivation regulation was the least internalized (amotivation).

Most studies have based their analyses on the assumption that health and well-being are part of more internal motivation [8]. However, according to our results, health is more linked to external regulation, while wellbeing is linked to internal regulation. These findings could partially explain why the main reason to quit was not systematically associated with quitting attempts and successful abstinence [8]. On the one hand, fear or worry may explain the desire to quit smoking, while on the other hand, realizing the benefit of abstinence explains the long-term change. Hence, fear-based programs tend to be less effective compared to those that stress the abstinence benefits and recognize small accomplishments [25].

On the basis of the SDT motivation framework, participants with more internalized regulation are more committed and persistent, and thus efficient in behavioral change [5]. As participants’ main motivation towards smoking cessation was intrinsic (58%) followed by identified regulation (24%), the lowest dropout after the first use of the mobile app (S0) and the end of the preparation program (S8) was expected from them. However, there is no clear trend of abandonment depending on the internalization degree of smoking cessation, and thus no concurrent validity. These results can be explained by the online intervention and the high dropout rate after the first use of the health-enhancing apps (43%) [26]. In addition, traditional smoking cessation interventions have dropout rates ranging from 10.8% to 77% [27].

### Limits and Future Directions

To ensure generalizability to other samples and context, this scale needs further study. Factor structures can differ across racial or ethnic samples in theoretically meaningful ways and the context in which the scale could be presented [28]. For this matter, psychometric studies remain an important part of research in the testing of psychological theories. The F-SCMS scale should be validated with a nonvirtual sample and include sociodemographic characteristics such as smoking history and measures of global motivation to change. For example, dependency severity was related to introjected regulation, where higher severity of guilt and shame drive treatment seeking [9]. To assess the predictive validity, it is also important to measure the relationship between the long-term abstinence rate and each motivation of the continuum of the SDT framework. These limitations will be addressed in a prospective study that has been validated by a French ethics committee (southeast), and allows for the collection of personal and health data [29].

As mentioned before, internalization is linked with successful withdrawal [18]. The question that remains to be answered is: to what extent can mobile apps enhance the core elements of the internalization process (autonomy, competence, and relatedness) in the context of smoking cessation?

For instance, studies showed that gamified mobile apps increase feelings of self-efficacy (i.e., the individual’s belief in their capacity to stop smoking) [30]. There are different game-based functionalities used as feedback of success or progress, goal setting (unlock badges, levels, or challenges), and social feedback [31] that positively reinforces engagement with the mobile app and the change process [25].

On the basis of the principles of positive or negative conditioning, it remains to be seen how the use of gamification within a mobile application could compromise autonomy since, on the one hand, rewards or punishments are external reinforcement. However, on the other hand, autonomy can be increased by supporting users’ initiatives, providing them with relevant information, and minimizing the feeling of pressure during the use of the mobile application.

Competence is a sense of mastery, and the ability to succeed and fulfillment [5]. This can be achieved by providing optimal challenges and offering success feedback focused on the user’s internal control and opportunities for growth. Autonomy and competence are associated with changes in tabaco use and long-term tabaco abstinence for adults [15,32]. The sense of belonging and connectedness could be enhanced through the social features offered by some mobile applications, facilitating the search for social validation when experiencing difficulties at the time of change, and facilitating the sharing of support from people who have managed to sustain such change.

## 5. Conclusions

Despite some limitations and unexpected findings, this was the first study to assess the degree of smoking cessation internalization in a large sample of French-speaking smokers who had enrolled in a preparation program using a mobile application. The F-SCMS shows consistent results with SDT framework and convergent validity.

The sole purpose of the study was the validation of the F-SCMS. After its validation, the F-SCMS was translated into Spanish and English as part of the smoking cessation preparation program offered by the mobile app Kwit SAS. The entire program was translated into the two languages by an official translator. In the hope that they will be used for research purposes and in future clinical settings, these versions are shared supplementary materials.

## Figures and Tables

**Table 1:** Summary of the frequency of main reasons for quitting by motivation towards smoking cessation.

Motivation Type			Reasons for Quitting (%, *n*)									
	Full Sample		Health		Money		Well-Being		Family		Child Project	
	*n*	%	*n*	%	*n*	%	*n*	%	*n*	%	*n*	%
Amot	264	2	74	28	111	42	36	14	16	6	27	10
Exter	1057	7	366	35	336	32	112	16	187	18	56	5
Introj	295	2	151	51	36	12	42	14	45	15	21	7
Ident	3814	24	1697	44	893	23	839	22	192	5	193	5
Integ	560	4	249	44	114	20	79	14	87	16	31	6
Intrin	8756	58	4014	46	1917	22	1895	22	537	6	393	4
Mixed	464	3	230	50	94	20	76	16	39	8	25	5
Total	15,210		6781	45	3501	23	3079	20	1103	7	746	5

Amot = amotivation; Exter = external; Introj = introjected; Ident = identified; Integ = integrated; Intrin = intrinsic.

**Table 2:** Alpha coefficients (diagonal) and Pearson correlations (lower triangle) between subscales of the final version of F-SCMS scale.

Subscales	Amot	Exter	Introj	Ident	Iteng	Intrins
Amot	0.80					
Exter	0.27	0.74				
Introj	0.23	0.54 *	0.74			
Ident	0.32	0.43 *	0.35	0.74		
Integ	0.25	0.41	0.31	0.67 **	0.78	
Intrin	0.32	0.46	0.32	0.60 *	0.65 *	0.73

Amot = amotivation; Exter = external; Introj = introjected; Ident = identified; Integ = integrated; Intrin = intrinsic. * *p* < 0.05, ** *p* < 0.001.

**Table 3:** Pearson correlations coefficients between subscales of the final version of F-SCMS scale and willingness, ability, and readiness to quit.

Sample		Subscales Scores					
*n* = 15,211	Mean (SD)	Amot	Exter	Introj	Ident	Iteng	Intrins
Willingness	7.2 (0.7)	0.07 **	0.24 **	0.19 **	0.39 **	0.37 **	0.32 **
Ability	5.4 (0.7)	0.01 **	0.07	0.01 **	0.1 **	0.15 **	0.16 **
Readiness	6.5 (0.54)	0.02 *	0.18 **	0.18 **	0.09 **	0.27 **	0.25 **

Amot = amotivation; Exter = external; Introj = introjected; Ident = identified; Integ = integrated; Intrins = intrinsic. * *p* < 0.05, ** *p* < 0.001.

## Data Availability

Data is not publicly available due to privacy policy. Authors can share summarized data by e-mail contact at: luz.bustamante93@parisnanterre.fr.

## Supplementary Materials

The following supporting information can be downloaded at: https://www.mdpi.com/article/10.3390/ejihpe12080082/s1, Table S1: The Spanish version of the Smoking Cessation Motivation Scale; Table S2: English version of the Smoking Cessation Motivation Scale.

## Appendix A

**Table A1:** French version of the Smoking Motivation Scale.

Lisez Attentivement Chacun des éléments Suivants. Indiquez dans Quelle Mesure Ils Correspondent à Votre état D’esprit Actuel. 1-pas du Tout D’accord à 5 Tout à Fait D’accord		Scale				
		1	2	3	4	5
Amot1	Je me demande si c’est le bon moment pour changer.					
Ext1	Je préfère utiliser mon argent pour en profiter avec mes proches.					
Intro1	Mes proches font pression sur moi pour que je change					
Ident1	Je veux améliorer d’autres aspects de ma vie.					
Integ1	Je veux vivre en accord avec mes principes les plus profonds.					
Intrin1	Je suis satisfait quand je fais un pas vers le changement.					
Amot2	J’ai d’autres priorités.					
Ext2	Je veux que mes proches soient fiers de moi					
Intro2	Je veux soulager les soucis que mes proches se font pour moi.					
Ident2	J’aimerais apporter des changements à ma situation actuelle.					
Integ2	Je veux améliorer mon mode/style de vie.					
Intrin2	Je trouve plaisante l’idée de mieux me connaître moi-même.					
Amot3	J’ai besoin d’un projet concret pour me motiver à changer.					
Ext3	Je veux avoir plus de temps à consacrer à mes proches.					
Intro3	Je me sentirais coupable de ne rien faire pour apporter des solutions à mes problèmes.					
Ident3	Cela m’aiderait à atteindre d’autres objectifs.					
Integ3	Je choisis de prendre plus soin de moi et de ma santé.					
Intrin3	Je trouve intéressant d’apprendre que je peux m’améliorer.					

The French smoking cessation motivation scale is validated by Luz Bustamante and Pr. Lucia Romo (2020) as part of a 9-step preparation program proposed by Kwit SAS.
