# Supplementary material for: Validation of the French Smoking Cessation Motivation Scale with French Smokers Using a Mobile App for Smoking Cessation
Source: Eur J Investig Health Psychol Educ. 2022 Aug 19;12(8):1179–90. doi: 10.3390/ejihpe12080082 (PMC9407113; doi:10.3390/ejihpe12080082)
Supplement: Supplementary file 1 [file ejihpe-12-00082-s001.zip › ejihpe-1821003-supplementary.pdf]

**Table S1:** Spanish Smoking Cessation Motivation Scale (S-SCMS)

| Lee atentamente cada una de las siguientes afirmaciones e indica en qué medida se corresponden con tu estado de ánimo actual. Tienes 5 niveles de respuesta: 1-Muy en desacuerdo y 5- Muy acuerdo | | Scale | | | | |
|---|---|---|---|---|---|---|
| | | 1 | 2 | 3 | 4 | 5 |
| Amot1 | Me pregunto si ahora es un buen momento para cambiar. | | | | | |
| Ext1 | Prefiero utilizar mi dinero para disfrutar con mis seres queridos. | | | | | |
| Intro1 | Mi familia y/o amigos me presionan para que cambie. | | | | | |
| Ident1 | Quiero mejorar otros aspectos de mi vida | | | | | |
| Integ1 | Quiero vivir de acuerdo con mis valores y principios. | | | | | |
| Intrin1 | Me siento satisfecho(a) cuando doy un paso hacia el cambio. | | | | | |
| Amot2 | Tengo otras prioridades. | | | | | |
| Ext2 | Quiero que mis seres queridos se sientan orgullosos de mí. | | | | | |
| Intro2 | Quiero aliviar las preocupaciones que mis familiares y/o amigos tienen por mí. | | | | | |
| Ident2 | Me gustaría hacer cambios en mi situación actual. | | | | | |
| Integ2 | Quiero mejorar mi estilo de vida. | | | | | |
| Intrin2 | Me gusta la idea de conocerme mejor. | | | | | |
| Amot3 | Necesito un proyecto concreto que me motive a cambiar. | | | | | |
| Ext3 | Quiero pasar más tiempo con mis seres queridos. | | | | | |
| Intro3 | Me sentiría culpable si no hiciera nada para solucionar mis problemas. | | | | | |
| Ident3 | Me ayudaría a lograr otros objetivos. | | | | | |
| Integ3 | Elijo cuidar mejor de mí mismo(a) y de mi salud. | | | | | |
| Intrin3 | Me parece interesante saber que puedo mejorar. | | | | | |

The French smoking cessation motivation scale is validated by Luz Bustamante and Pr. Lucia Romo (2020) as part of a 9-step preparation program proposed by Kwit SAS.

**Table S2:** English Smoking Cessation Motivation Scale (E-SCMS)

Motivation is a driving force that pushes you to act. It is like a wind that moves the ship of change. It gives you the direction and strength with which you move forward!

| Carefully read each of the following statements and indicate to what extent they correspond to your current state of mind. You have 5 levels of response: 1- Strongly disagree to 5-Strongly agree | | Scale | | | | |
|---|---|---|---|---|---|---|
| | | 1 | 2 | 3 | 4 | 5 |
| Amot1 | I'm wondering if it's the right time to change. | | | | | |
| Ext1 | I would rather spend my money on things I enjoy with my loved ones. | | | | | |
| Intro1 | My loved one's pressure me to change. | | | | | |
| Ident1 | I want to improve other aspects of my life. | | | | | |
| Integ1 | I want to live in accordance with my core beliefs. | | | | | |
| Intrin1 | I am satisfied when I take a step towards change. | | | | | |
| Amot2 | I have other priorities. | | | | | |
| Ext2 | I want my loved ones to be proud of me | | | | | |
| Intro2 | I want to avoid my loved ones' worries about me. | | | | | |
| Ident2 | I want to make changes to my current situation. | | | | | |
| Integ2 | I want to improve my lifestyle. | | | | | |
| Intrin2 | I like the idea of getting to know myself better. | | | | | |
| Amot3 | I need a concrete project to encourage me to change. | | | | | |
| Ext3 | I want to have more time to spend with my loved ones. | | | | | |
| Intro3 | I would feel guilty if I didn't do anything to solve my problems. | | | | | |
| Ident3 | It would help me to achieve other goals. | | | | | |
| Integ3 | I choose to take better care of myself and my health. | | | | | |
| Intrin3 | I find it interesting to learn that I can improve. | | | | | |

The French smoking cessation motivation scale is validated by Luz Bustamante and Pr. Lucia Romo (2020) as part of a 9-step preparation program proposed by Kwit SAS.
